# Recommendations for extracorporeal membrane oxygenation (ECMO) in COVID-19 patients: Consensus paper of the Medical University of Vienna

**DOI:** 10.1007/s00508-020-01708-8

**Published:** 2020-07-03

**Authors:** Dominik Wiedemann, Martin H. Bernardi, Klaus Distelmaier, Georg Goliasch, Christian Hengstenberg, Alexander Hermann, Michael Holzer, Konrad Hoetzenecker, Walter Klepetko, György Lang, Andrea Lassnigg, Günther Laufer, Ingrid A. M. Magnet, Klaus Markstaller, Martin Röggla, Bernhard Rössler, Peter Schellongowski, Paul Simon, Edda Tschernko, Roman Ullrich, Daniel Zimpfer, Thomas Staudinger, COVID-19 ECMO Research Group Medical University of Vienna

**Affiliations:** 1 Division of Cardiac Surgery, University Department of Surgery, Vienna General Hospital, Medical University of Vienna, Währinger Gürtel 18–20, 1090 Vienna, Austria; 2 Division of Cardiac Thoracic Vascular Anesthesia and Intensive Care Medicine, University Department of Anaesthesia, Intensive Care Medicine and Pain Medicine, Medical University of Vienna, Vienna, Austria; 3 Division of Cardiology, University Department of Medicine II, Medical University of Vienna, Vienna, Austria; 4 University Department of Medicine I, Vienna General Hospital, Medical University of Vienna, Währinger Gürtel 18–20, 1090 Vienna, Austria; 5 University Department of Emergency Medicine, Medical University of Vienna, Vienna, Austria; 6 Division of Thoracic Surgery, University Department of Surgery, Medical University of Vienna, Vienna, Austria; 7 Medical Simulation and Emergency Management Research Group, Medical University of Vienna, Vienna, Austria; 8 Division of General Anaesthesia and Intensive Care Medicine, University Department of Anaesthesia, Intensive Care Medicine and Pain Medicine, Medical University of Vienna, Vienna, Austria

**Keywords:** ECMO, COVID-19, Extracorporeal life support, Pandemic, Corona virus

## Abstract

The pandemic from the SARS-CoV‑2 virus is currently challenging healthcare systems all over the world. Maintaining appropriate staffing and resources in healthcare facilities is essential to guarantee a safe working environment for healthcare personnel and safe patient care. Extracorporeal membrane oxygenation (ECMO) represents a valuable therapeutic option in patients with severe heart or lung failure. Although only a limited proportion of COVID-19 patients develop respiratory or circulatory failure that is refractory to conventional treatment, it is of utmost importance to clearly define criteria for the use of ECMO in this steadily growing patient population. The ECMO working group of the Medical University of Vienna has established the following recommendations for ECMO support in COVID-19 patients.

## Background

The pandemic from the SARS-CoV‑2 virus is currently challenging health care systems all over the world [1]. Although many COVID-19 patients only show moderate symptoms, a subpopulation develops severe respiratory failure such as acute respiratory distress syndrome (ARDS) [2, 3]. Moreover, cardiac involvement leading to fulminant myocarditis, arrhythmia, and circulatory failure has been described [3, 4]. Once COVID-19 patients require mechanical ventilation, mortality rates increase dramatically.

Despite the fact that in the USA, Spain and Italy (the countries with the highest numbers of COVID-19 patients at the time this document was created) the number of ECMO patients among COVID-19 cases is low, it seems of outmost importance to address this issue in Austria and Vienna by establishing recommendations for ECMO support in COVID-19 patients.

For additional/general information on this topic please refer to the guidelines of the Extracorporeal Life Support Organization (ELSO) [5].

## Allocation

### Should ECMO treatment be considered for COVID-19 patients?

Whereas in more affected countries triaging of patients to give treatment priority to those with more chances of survival has already become standard practice, triage is not necessary in Austria so far. Nevertheless, indications for ECMO support in COVID-19 should be defined restrictively under consideration of possible future resource shortages [6], which does not generally imply an exclusion of COVID-19 patients from ECMO treatment; however, advanced age, comorbidities and conditions limiting prognosis in general are important factors, which have to be taken into consideration before initiation of ECMO support in COVID-19 patients.

If the situation in Austria changes and a triage is necessary similar to Italy, Spain and other countries, the decision for ECMO support will be made according to the available resources, especially with respect to availability of intensive care unit (ICU) beds and mechanical ventilation.

The number of hospitals offering ECMO is increasing steadily [7]; however, the start of novel ECMO programs is currently not recommended as high volume and profound clinical experience are important factors of clinical outcome [5, 8]. Moreover, the infection risk for staff members is definitely lower when an experienced team is available. Of note: safety first. Especially in a pandemic, the protection of healthcare workers is of outmost importance and has to be prioritized even over the patient’s condition.

### ECMO for non-COVID-19 patients during the pandemic

Since Austria is currently not in need of triage, the decision regarding ECMO implementation should be taken according to the algorithms prior to the pandemic; however, if the COVID-19 pandemic leads to significant resource shortages it might be necessary to make more restrictive decisions also for non-Covid-19 patients.

### Patient prioritization

Especially during the pandemic, patient prioritization is crucial. First come—first served motivated decisions should be avoided due to resource consumption of patients with worse prognosis, potentially resulting in shortages for those with better prognosis. This is of outmost importance because present decisions may have significant impact on future resource availability. Young patients without comorbidities should have the highest priority.

With dynamic changes of capacities during the course of the pandemic, prioritization should be adapted and changed accordingly. Even adaptation of decision pathways from one shift to another seems possible in extreme situations.

## Standard operating procedures and exclusion criteria of COVID-19 positive ECMO candidates

If a SARS-CoV‑2 positive patient is judged as potential ECMO candidate outside of the Vienna General Hospital (AKH-Wien) according to given criteria, the AKH intensive care coordinator (an experienced intensivist with 24/7 availability) is the first to contact.

### Personal protective equipment (PPE)

For ECMO implementation, we recommend to follow the general guidelines of the Vienna Clinic Network (Wiener Krankenhausverbund, KAV) for personal protection in COVID-19 patients [9]. Additional recommendations for the surgeon to avoid wound infections are to use respirator face masks (FFP‑2 or FFP-3) without valves. Alternatively, respirator face masks with valve may be used in combination with standard medical masks.

### Exclusion criteria for ECMO implementation in COVID-19 patients

Exclusion criteria may dynamically change according to resources and capacities. From current perspective, the criteria listed in Table 1 should be used to either exclude or critically evaluate patients for ECMO support.

**Table 1:** Contraindications for ECMO support in COVID-19 patients

Absolute contraindications	Relative contraindications
Rejection by the patient	Age >65 years^a^ (depending on the biological age)
Pre-existing severe neurological deficit, advanced dementia	Ventilation duration prior ECMO >7 days
End-stage disease (life expectancy <1 year)	Relevant immunosuppressive therapies
Known severe brain injury	Systemic hematologic disorders
Age >75 years or age >70 plus ≥2 relative contraindications^a^	Additional organ failure (except kidney)
End-stage lung disease	Frailty [10]
Disseminated malignancy	Severe aortic regurgitation (VA ECMO)
Child-Pugh C liver cirrhosis	Severe peripheral vascular disease (VA ECMO)
<1 year after allogeneic stem cell transplantation	Chronic heart failure NYHA IV (without option for heart transplantation or ventricular assist device)

^a^Age limits possibly will have to be adapted according to the course of the pandemic

*NYHA* New York Heart Association

## Venovenous ECMO (respiratory ECMO)

### Principle

The use of ECMO in COVID-19 is a rescue treatment, if mechanical ventilation cannot guarantee appropriate gas exchange anymore, resulting in life-threatening or organ-damaging hypoxia and/or hypercapnia; however, ECMO therapy is also indicated when the negative effects of invasive mechanical ventilation are considered unacceptable. Timing of ECMO support and patient selection are crucial for outcome.

#### Indications and contraindications

**Fig. 1:**
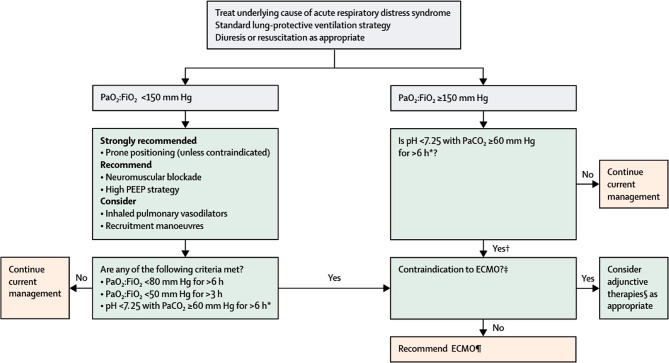
Algorithm for management of acute respiratory distress syndrome (ARDS) ([5, 11] from [12]). *With respiratory rate increased to 35 breathes per minute and mechanical ventilation settings adjusted to keep a plateau airway pressure of ≤ 35 cm of water. †Consider neuromuscular blockade, ‡There are no absolute contraindications that are agreed upon except end-stage respiratory failure when lung transplantation will not be considered, §Eg, neuromuscular blockade, high PEEP strategy, inhaled pulmonary vasodilatators, recruitment manoeuvres, high frequency oscillatory ventilation, ¶Recommend early consideration of ECMO. Depending on progress of patient’s clinical condition, presentation for ECMO should already be considered in case of progressive respiratory deterioration (PaO2/FiO2 Ratio of < 150)

The Extracorporeal Life Support Organization has published a standardized algorithm for the management of ARDS (Fig. 1; [5, 11]). This algorithm may be of aid to clinicians to evaluate ECMO support in COVID-19 patients under conditions of optimized respiratory treatment. In order to prevent secondary organ damage in time, we recommend early consideration of ECMO treatment. Depending on progress of patient’s clinical condition, presentation for ECMO should already be considered in case of progressive respiratory deterioration (PaO2/FiO2 ratio of <150).

##### Comments

- Absolute contraindications refer explicitly to patients with severe lung failure due to present SARS-CoV‑2 infection.
- Relative contraindications describe factors associated with a worse outcome in patients with need for respiratory ECMO. In case of present relative contraindications, ECMO treatment should only be applied in exceptional cases!
- For each point, individual assessment is needed.
- The presence of multiple relative contraindications worsens the prediction of patient outcome substantially.
- Relative contraindications are evaluated in accordance to resources and capacities.
- Ideal ECMO candidates would be patients <65 years without pre-existing diseases and single organ failure (lung).

#### Cannulation

- We recommend a cannulation strategy with greatest experience for each center, for example: femorojugular configuration.
- Cannula size is to be selected according to the expected blood flow demand (goal: ECMO blood flow >60% of the patient’s cardiac output).
- COVID-19 patients qualifying for ECMO are those with the most severe lung failure (rescue ECMO), therefore almost complete lung replacement is to be expected, therefore we recommend to choose a bigger drainage cannula (in normal sized adults with ultrasound assessed venous diameter of ≥10 mm at least 23 F, ideally 25 F).
- The perfusion cannula chosen is usually 2–4 F smaller in diameter.

#### Ventilation strategy in VV ECMO

In VV ECMO, a lung protective ventilation strategy is recommended including reduction of tidal volumes, driving pressure and respiratory rate [13–15]. The PEEP titration should be adjusted individually according to the recruitment potential [16].

## Veno-arterial ECMO in COVID-19 patients (cardiac ECMO)

Although in COVID-19 patients primarily pulmonary failure is expected, there may be numerous patients requiring VA ECMO support. The underlying causes may be the development of consecutive circulatory failure and also potential primary cardiac involvement with arrythmias or myocarditis. Therefore, in addition to the common hemodynamic monitoring, echocardiography as part of ECMO evaluation in COVID-19 patients is necessary in order to enable the choice between VV and VA ECMO accordingly; however, it has to be pointed out that in pulmonary failure and possibly even high dose vasopressors, but otherwise more or less preserved left and right ventricle function, VA ECMO is not necessarily indicated.

### Choice of cannulation strategy

As in non-COVID-19 patients, the cannulation strategy needs to be adapted to the individual clinical scenario. In patients with cardiogenic shock or during resuscitation, percutaneous implantation via the femoral artery and vein is recommended.

An additional cannula for distal leg perfusion is strongly advised in all cases. Due to the fact that most COVID-19 patients have respiratory complications, insufficient oxygenation of the body’s upper half based on the Harlequin effect is likely [17]. Therefore, in case of femoro-femoral VA ECMO, oxygenation of the body’s upper half needs to be monitored closely by saturation measurement at the right upper limb. In the case of insufficient oxygenation, an early change of the ECMO set-up is recommended. Especially in COVID-19 patients, an upgrading to V-VA ECMO is the primary option to keep a potential contagion risk as low as possible. For V-VA upgrade in such a case an additional cannula is introduced into the jugular vein, which is connected then to the arterial (returning) line. Alternatively, changing the arterial cannulation configuration from femoral to subclavian (axillary) artery is possible; however, as this option is tied to surgical capacities, it involves more healthcare workers and therefore has the higher risk of contagion.

Transesophageal echocardiography (TEE) guided cannula placement is the most established and common imaging modality in our hands; however, TEE is an aerosol-forming procedure, and the advantages need to be balanced against a potential risk of infection. Transthoracic and abdominal ultrasound imaging may be used alternatively.

### Circulatory failure in VV ECMO

Some patients with primarily respiratory failure develop right heart failure (ARDS, high PEEP) or left heart failure (myocarditis) during VV ECMO. Therefore, regular monitoring by echocardiography, at least once daily is recommended. In case of evolving heart failure, upgrade or change of the VV ECMO is necessary. The primary choice again is to switch to V-VA ECMO with an additional return cannula inserted in the femoral artery. The second option would be to switch from VV to VA ECMO by cannulation of the subclavian (axillary) artery.

The final decision which approach to choose, is based on the available vascular access as well as the patient’s condition and needs to be adjusted to the experience of the individual team on duty.

### Special clinical scenario: extracorporeal cardiopulmonary resuscitation (eCPR)

The Vienna General Hospital runs one of the largest eCPR programs in Europe. While indications and patient selection for eCPR are crucial in general, during a pandemic the available resources and the risk of contagion of healthcare workers are additional factors. The Department of Emergency Medicine of the Medical University of Vienna has established criteria for eCPR in non-COVID-19 patients as illustrated in Table 2.

**Table 2:** Criteria for extracorporeal cardiopulmonary resuscitation (eCPR) to facilitate an uncomplicated assessment at an early stage of emergency medical service effort [18]

Aim: Time from cardiac arrest to hospital admission <60 min, if
Witnessed cardiac arrest
Bystander resuscitation or first medical contact <5 min
Age under 70 years
Shockable initial rhythm or return of spontaneous circulation at any time during resuscitation
Body mass index <35
A persistant end tidal carbon dioxide >14 mm Hg
Pupils not anisocoric/unequal/mydriatic
No end-stage disease
No severe peripheral vascular disease

The risk of contagion needs to be assumed as critical for healthcare workers especially in an eCPR setting (aerosol-generating procedure). During eCPR of SARS-CoV‑2 positive patients, early (prehospital) contact of the emergency team is important, to enable an out of hospital decision for or against ECMO implementation. Transport of SARS-CoV‑2 positive patients to the hospital for eCPR, just to judge them unsuitable for ECMO should be avoided. Healthcare workers would be put at risk for contagion without any prospect of successful treatment.

In case of eCPR of patients with unknown SARS-CoV‑2 status (which will most probably be the case), the procedure needs to be performed as if the patients would be SARS-CoV‑2 positive (PPE!).

## Discontinuation of ECMO for futility and end-of-life decisions

Patients with ECMO in general should be evaluated on a daily basis. During the whole course of the patient PPE needs to be followed. In case of pulmonary and/or cardiac recovery the routine weaning protocol should be followed.

In case of no recovery, even COVID-19 patients should be evaluated for further (permanent) treatment (transplantation or ventricular assist devices). Although, from the current point of view, it is rather unlikely that many COVID-19 will qualify for such a treatment, this option should be evaluated especially in younger patients. Based on recent a publication from our university, we know that the patient’s survival chances decrease already after the 7th day on VA ECMO support. This does not mean to stop any ECMO support by day 7, but treatment goals should be re-evaluated after 1 week of ECMO support and further decisions adjusted accordingly [19, 20].

If there are no options for further treatment modalities, discontinuation of ECMO support needs to be discussed. In respiratory ECMO, a subgroup of patients may recover even after significantly longer support times; therefore, an individual decision to continue ECMO support may be justified (by taking the previous course of organ failure and additional organ failure into account) [21, 22]. These decisions will be dynamically affected by resource shortages and capacities during the pandemic as well.

## References

[1] Grasselli G, Pesenti A, Cecconi M. Critical care utilization for the COVID-19 outbreak in Lombardy, Italy: Early experience and forecast during an emergency response. JAMA. 2020;323(16):1545–6. 10.1001/jama.2020.4031. 10.1001/jama.2020.4031 32167538

[2] Wang D, Hu B, Hu C, Zhu F, Liu X, Zhang J, Wang B, Xiang H, Cheng Z, Xiong Y (2020). Clinical characteristics of 138 hospitalized patients with 2019 novel coronavirus-infected pneumonia in Wuhan, China. JAMA.

[3] Huang C, Wang Y, Li X, Ren L, Zhao J, Hu Y, Zhang L, Fan G, Xu J, Gu X (2020). Clinical features of patients infected with 2019 novel coronavirus in Wuhan, China. Lancet.

[4] Hu H, Ma F, Wei X, Fang Y (2020). Coronavirus fulminant myocarditis saved with glucocorticoid and human immunoglobulin. Eur Heart J.

[5] ELSO. ELSO guidance document: ECMO for COVID-19 patients with severe cardiopulmonary failure. ASAIO J. 2020;66(5):472–4. 10.1097/MAT.0000000000001173. 10.1097/MAT.0000000000001173 PMC7273858 32243267

[6] Ramanathan K, Antognini D, Combes A, Paden M, Zakhary B, Ogino M, MacLaren G, Brodie D, Shekar K (2020). Planning and provision of ECMO services for severe ARDS during the COVID-19 pandemic and other outbreaks of emerging infectious diseases. Lancet Respir Med.

[7] Organization ELS (2020). ECLS registry report.

[8] Banjas N, Hopf HB, Hanisch E, Friedrichson B, Fichte J, Buia A (2018). ECMO-treatment in patients with acute lung failure, cardiogenic, and septic shock: mortality and ECMO-learning curve over a 6-year period. J Intensive Care.

[9] Wiener Krankenanstaltenverbund. Schutzbekleidung bei Corona Virus SARS-COV-2/COVID-19. 2020. https://info.wienkav.at/. Last access: March 2020.

[10] De Geer L, Fredrikson M, Tibblin AO (2020). Frailty predicts 30-day mortality in intensive care patients: A prospective prediction study. Eur J Anaesthesiol.

[11] Combes A, Hajage D, Capellier G, Demoule A, Lavoue S, Guervilly C, Da Silva D, Zafrani L, Tirot P, Veber B (2018). Extracorporeal membrane oxygenation for severe acute respiratory distress syndrome. N Engl J Med.

[12] Abrams D, Ferguson ND, Brochard L, Fan E, Mercat A, Combes A, Pellegrino V, Schmidt M, Slutsky AS, Brodie D (2019). Lancet Respir Med.

[13] Papazian L, Aubron C, Brochard L, Chiche JD, Combes A, Dreyfuss D, Forel JM, Guerin C, Jaber S, Mekontso-Dessap A (2019). Formal guidelines: management of acute respiratory distress syndrome. Ann Intensive Care.

[14] Fan E, Del Sorbo L, Goligher EC, Hodgson CL, Munshi L, Walkey AJ, Adhikari NKJ, Amato MBP, Branson R, Brower RG (2017). An official American Thoracic Society/European Society of Intensive Care Medicine/Society of Critical Care Medicine clinical practice guideline: mechanical ventilation in adult patients with acute respiratory distress syndrome. Am J Respir Crit Care Med.

[15] Serpa Neto A, Schmidt M, Azevedo LC, Bein T, Brochard L, Beutel G, Combes A, Costa EL, Hodgson C, Lindskov C (2016). Associations between ventilator settings during extracorporeal membrane oxygenation for refractory hypoxemia and outcome in patients with acute respiratory distress syndrome: a pooled individual patient data analysis: mechanical ventilation during ECMO. Intensive Care Med.

[16] Schmidt M, Pellegrino V, Combes A, Scheinkestel C, Cooper DJ, Hodgson C (2014). Mechanical ventilation during extracorporeal membrane oxygenation. Crit Care.

[17] Lotz C, Ritter O, Muellenbach RM (2014). Assisted beating of the ischemic heart: how to manage the pulseless ST—Segment-elevation myocardial infarction patient. Circulation.

[18] Poppe M, Schriefl C, Steinacher A, Clodi C, Warenits AM, Nürnberger A, Hubner P, Holzer M, Horvat J, Wiedemann D (2020). Extracorporeal cardiopulmonary resuscitation at the emergency department: a retrospective patient selection evaluation. Eur J Anaesthesiol.

[19] ELSO. ELSO general guidelines for all ECLS cases. 2017. https://www.elso.org. Last access: August 2017.

[20] Pichler P, Antretter H, Dunser M, Eschertzhuber S, Gottardi R, Heinz G, Pölzl G, Pretsch I, Rajek A, Wasler A (2015). Use of ECMO in adult patients with cardiogenic shock: a position paper of the Austrian Society of Cardiology. Wien Klin Wochenschr.

[21] Posluszny J, Rycus PT, Bartlett RH, Engoren M, Haft JW, Lynch WR, Park PK, Raghavendran K, Napolitano LM (2016). Outcome of adult respiratory failure patients receiving prolonged (≥14 days) ECMO. Ann Surg.

[22] Camboni D, Philipp A, Lubnow M, Bein T, Haneya A, Diez C, Schmid C, Müller T (2011). Support time-dependent outcome analysis for veno-venous extracorporeal membrane oxygenation. Eur J Cardiothorac Surg.

